# The efficiency of bridging sheet recruitment determines HIV-1 R5 envelope sensitivity to soluble CD4 and macrophage tropism

**DOI:** 10.1186/1742-4690-9-S2-P141

**Published:** 2012-09-13

**Authors:** O O'Connell, A Repik, JD Reeves, MP Gonzalez-Perez, B Quitadamo, M Duenas-Decamp, P Peters, R Lin, ED Anton, S Zolla-Pazner, D Corti, A Wallace, S Wang, X Kong, S Lu, PR Clapham

**Affiliations:** 1 Monogram Biosciences, San Francisco, CA, USA; 2 University of Massachusetts, Amherst, MA, USA; 3 New York University Langone School of Medicine, New York, NY, USA; 4 Humabs Biomed SA, Bellinzona, Switzerland; 5 New York University School of Medicine, New York, NY, USA; 6 University of Massachusetts Medical School, Worcester, MA, USA

## Background

HIV-1 R5 viruses vary extensively in their capacity to infect macrophages. R5 viruses that confer efficient infection of macrophages are able to exploit low levels of CD4 for infection and they predominate in brain tissue where macrophages are a major target for infection. HIV-1 R5 variants that are transmitted are generally non-macrophage-tropic. Here, we investigated the sensitivity of macrophage-tropic and non-macrophage-tropic R5 envelopes to neutralizing antibodies.

## Methods

Env+ pseudovirion neutralization assays were carried out using HeLa TZM-bl cells. Envelope capture ELISAs assessed monoclonal antibody binding to monomeric gp120.

## Results

We observed striking differences in the sensitivity of Env+ pseudovirions to soluble CD4 compared to neutralizing monoclonal antibodies that target the CD4 binding site. Macrophage-tropic R5 envelopes were sensitive to sCD4, while non-macrophage-tropic envelopes were significantly more resistant. In contrast, all envelopes were sensitive to VRC01 regardless of tropism, while mab b12 conferred an intermediate neutralization pattern with all the macrophage-tropic and about half of the non-macrophage-tropic envelopes sensitive.

## Conclusion

CD4, b12 and VRC01 share binding specificities on the outer domain of gp120. However, these reagents differ in their ability to induce conformational changes on the trimeric envelope and in specificity for residues on the V1V2 loop stem and β20-21 junction that are targets for CD4 in recruiting the bridging sheet. These different binding specificities of CD4, b12 and VRC01 likely explain the striking differences in envelope sensitivity to inhibition by these reagents. However, they also provide an insight into the envelope mechanisms that control macrophage-tropism. We present a model where the efficiency of bridging sheet recruitment by CD4 is a major determinant of HIV-1 R5 envelope sensitivity to soluble CD4 and macrophage tropism.

